# CTLA-4 Expression Is a Promising Biomarker of Idiopathic Pulmonary Arterial Hypertension and Allows Differentiation of the Type of Pulmonary Hypertension

**DOI:** 10.3390/ijms232415910

**Published:** 2022-12-14

**Authors:** Michał Tomaszewski, Paulina Małkowska, Olga Sierawska, Rafał Hrynkiewicz, Ewa Mroczek, Szymon Darocha, Anna Hymos, Piotr Błaszczak, Ewelina Grywalska, Paulina Niedźwiedzka-Rystwej

**Affiliations:** 1 Department of Cardiology, Medical University of Lublin, 20-954 Lublin, Poland; 2 Doctoral School, University of Szczecin, 71-412 Szczecin, Poland; 3 Institute of Biology, University of Szczecin, 71-412 Szczecin, Poland; 4 Department of Cardiology, Institute of Heart Diseases, Jan Mikulicz-Radecki University Teaching Hospital, 51-124 Wroclaw, Poland; 5 Department of Pulmonary Hypertension, Thromboembolic Diseases and Cardiology, Centre of Postgraduate Medical Education, 05-400 Otwock, Poland; 6 Department of Experimental Immunology, Medical University of Lublin, 20-093 Lublin, Poland; 7 Department of Cardiology, Cardinal Wyszynski Hospital, 20-718 Lublin, Poland

**Keywords:** CTLA-4, pulmonary arterial hypertension, lymphocytes

## Abstract

Pulmonary arterial hypertension (PAH) is an increasingly frequently diagnosed disease, the molecular mechanisms of which have not been thoroughly investigated. The aim of our study was to investigate subpopulations of lymphocytes to better understand their role in the molecular pathomechanisms of various types of PAH and to find a suitable biomarker that could be useful in the differential diagnosis of PAH. Using flow cytometry, we measured the frequencies of lymphocyte subpopulations CD4+CTLA-4+, CD8+ CTLA-4+ and CD19+ CTLA-4+ in patients with different types of PAH, namely pulmonary arterial hypertension associated with congenital heart disease (CHD-PAH), pulmonary arterial hypertension associated with connective tissue disorders (CTD-PAH), chronic thromboembolic pulmonary hypertension (CTEPH) and idiopathic pulmonary arterial hypertension (iPAH), and in an age- and sex-matched control group in relation to selected clinical parameters. Patients in the iPAH group had the significantly highest percentage of CD4+CTLA-4+ T lymphocytes among all PAH groups, as compared to those in the control group (*p* < 0.001), patients with CTEPH (*p* < 0.001), CTD-PAH (*p* < 0.001) and CHD-PAH (*p* < 0.01). In iPAH patients, the percentages of CD4+CTLA-4+ T cells correlated strongly positively with the severity of heart failure New York Heart Association (NYHA) Functional Classification (r = 0.7077, *p* < 0.001). Moreover, the percentage of B CD19+CTLA-4+ cells strongly positively correlated with the concentration of NT-proBNP (r = 0.8498, *p* < 0.001). We have shown that statistically significantly higher percentages of CD4+CTLA-4+ (*p* ≤ 0.01) and CD8+ CTLA-4+ (*p* ≤ 0.001) T cells, measured at the time of iPAH diagnosis, were found in patients who died within 5 years of the diagnosis, which allows us to consider both of the above lymphocyte subpopulations as a negative prognostic/predictive factor in iPAH. CTLA-4 may be a promising biomarker of noninvasive detection of iPAH, but its role in planning the treatment strategy of PAH remains unclear. Further studies on T and B lymphocyte subsets are needed in different types of PAH to ascertain the relationships that exist between them and the disease.

## 1. Introduction

Pulmonary arterial hypertension (PAH) is a severe clinical condition characterized by enhanced pulmonary vascular resistance (PVR), leading to increased pulmonary artery pressure (PAP)and the remodeling of the pulmonary arteries [1]. If left untreated, it leads to deterioration of right ventricular function, multiorgan failure and death. Invasive hemodynamic evaluation with right heart catheterization is the gold standard to establish the diagnosis of PAH. According to a recent update, pulmonary arterial hypertension (PAH) is diagnosed when the mean pulmonary artery pressure (mPAP) is ≥20 mm Hg and the normal pulmonary capillary wedge pressure (PCWP) is ≤15 mm Hg [2].

Consistent with the European Society of Cardiology (ESC)/European Respiratory Society (ERS) Guidelines, there are five groups of pulmonary hypertension (PH), according to clinical and pathophysiological criteria: group 1 refers to idiopathic pulmonary arterial hypertension (iPAH), as well as drug-induced PAH, connective tissue disease-related PAH and all heritable forms of PAH; group 2 includes the PH secondary to left-sided heart failure; group 3 includes PH due to the chronic lung disease and/or hypoxia; group 4 is called chronic thromboembolic pulmonary hypertension (CTEPH); group 5 consists of PAH due to uncertain multifactorial mechanisms [3,4].

Targeted medical therapy or interventional treatment can be offered to patients diagnosed with PAH and CTEPH, respectively. The prognosis of PAH varies broadly and depends mostly on the etiology of PAH, but is also based on hemodynamic, biochemical and functional parameters that indicate the severity of right ventricular failure, as well as on response to specific treatment. Risk stratification seems to be crucial for identifying patients at high risk and for optimizing therapeutic management. Thus, biomarkers and molecules may specifically indicate the disease and provide information about the disease stage and treatment response in a relatively easily accessible and noninvasive way.

CTLA-4 (cytotoxic t cell antigen 4) (CD152) molecules belong to the type I membrane receptor family and play an important role in signaling between immune cells [5]. CTLA-4 is mainly localized on the surface of activated CD4+ T cells and regulatory T cells (Treg), as well as on B19+ cells and dendritic cells [5,6]. The ligands of this receptor are CD80 and CD86 molecules, which are mostly seen on antigen-presenting cells. The main function of CTLA-4 is inhibitory; it is a key element in the negative regulation of the immune response, and when combined with a specific ligand, it inhibits T lymphocytes [7]. Two types of mechanisms influence this. The first is an extracellular mechanism and involves affecting the ability of antigen presenting cells (APCs) to stimulate T cells. The second mechanism is intracellular and involves suppression of signals sent to T cells [8]. The reproducibility of CTLA-4 measurements were shown by Grywalska et al. [9,10].

The aim of the present study was to investigate lymphocyte subpopulations and better understand their role in the molecular pathomechanisms of different types of PAH, and to find a new biomarker that could be useful and widely used in the differential diagnosis of PAH.

## 2. Results

**Figure 1:**
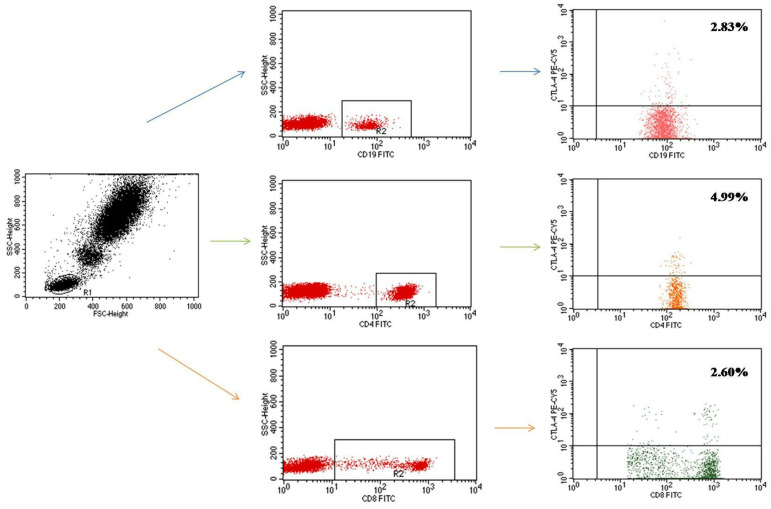
Evaluation of the percentage of lymphocytes (B CD19+, T CD4+, T CD8+) with CTLA-4 antigen expression in a patient with idiopathic pulmonary arterial hypertension. The signal confirming the presence of receptors on cells was present on 2.83% of CD19+ B cells, 4.99% of CD4+ T cells, and 2.60% of CD8+ T cells.

Cytometric analysis allowed us to determine the percentage of CD19+ B cells, CD4+ T cells and CD8+ T cells with CTLA-4 receptor expression (Figure 1).

**Figure 2:**
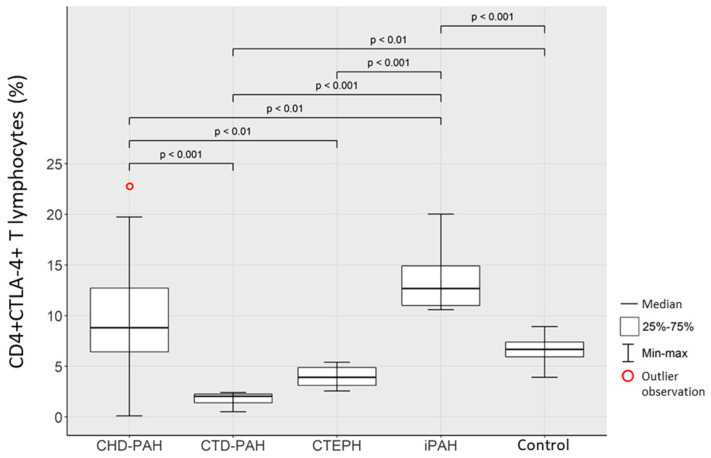
Percentage of CD4+CTLA4+ T lymphocytes in CHD-PAH, CTD-PAH, CTEPH, iPAH patients and control subjects.

Patients in the iPAH group had, by significant distance, the highest percentage of CD4+CTLA-4+ T lymphocytes among all PAH groups, as compared to those in the control group (*p* < 0.001), patients with CTEPH (*p* < 0.001), CTD-PAH (*p* < 0.001) and CHD-PAH (*p* < 0.01). Additionally, a higher percentage of CD4+CTLA4+ T lymphocytes was observed in CHD-PAH patients, as compared to CTD-PAH (*p* < 0.001) and CTEPH (*p* < 0.01) patients. The lowest percentage of CD4+CTLA4+ T lymphocytes was in the group of patients with CTD-PAH, which was statistically significant when compared to the control group (*p* < 0.01) (Table 1). The obtained relationships are presented in Figure 2.

**Figure 3:**
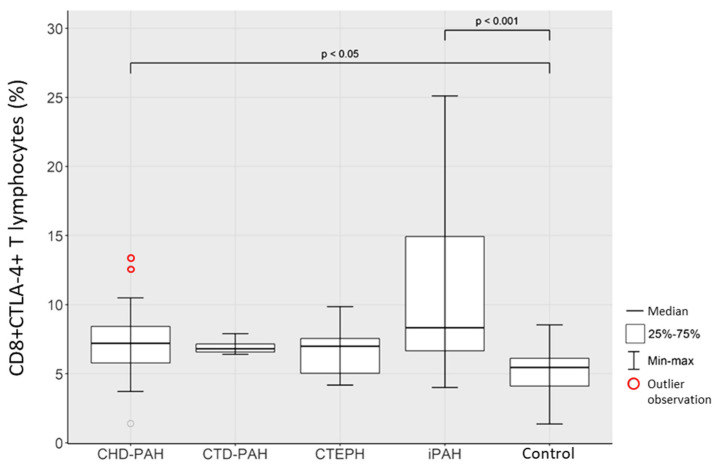
Percentage of CD8+CTLA4+ T lymphocytes in CHD-PAH, CTD-PAH, CTEPH, iPAH patients and control subjects.

Comparison of the percentage of CD8+CTLA4+ T lymphocytes in selected types of PAH and in the control group revealed a significantly higher percentage of these lymphocytes in the group of patients with iPAH than in the control group (*p* < 0.001). The obtained relationships are shown in Figure 3.

**Figure 4:**
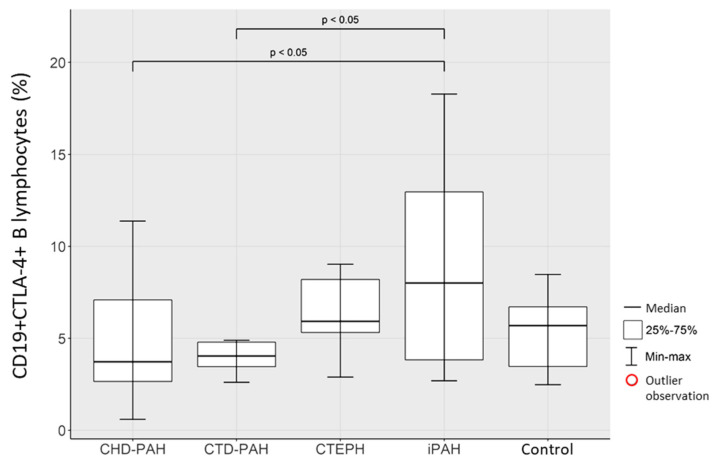
Percentage of CD19+CTLA4+ B lymphocytes in CHD-PAH, CTD-PAH, CTEPH, iPAH patients and control subjects.

Comparison of the percentage of CD19+CTLA4+ B lymphocytes in selected types of PAH and in the control group revealed a significantly higher percentage of these lymphocytes in the iPAH group than in the CTD-PAH and CHD-PAH groups (*p* < 0.05). The resulting relationships are shown in Figure 4.

**Figure 5:**
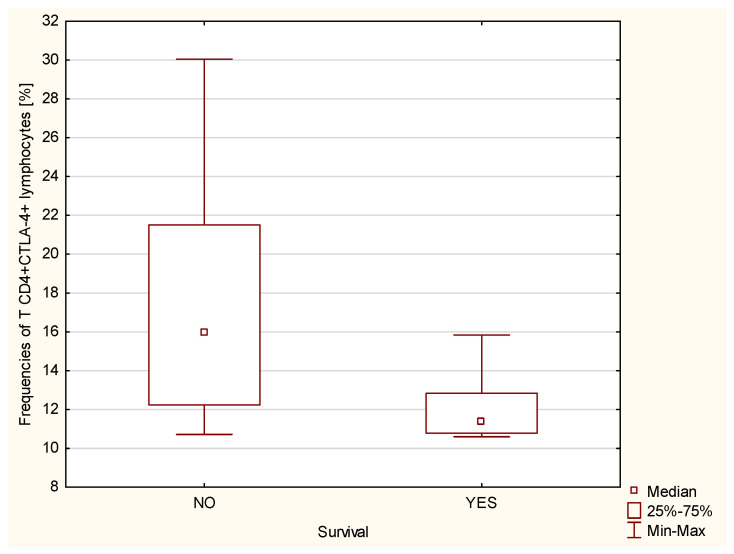
Percentage of CD4+CTLA4+ T lymphocytes in iPAH patients taking into account the survival of patients with iPAH (*p* ≤ 0.01).

**Figure 6:**
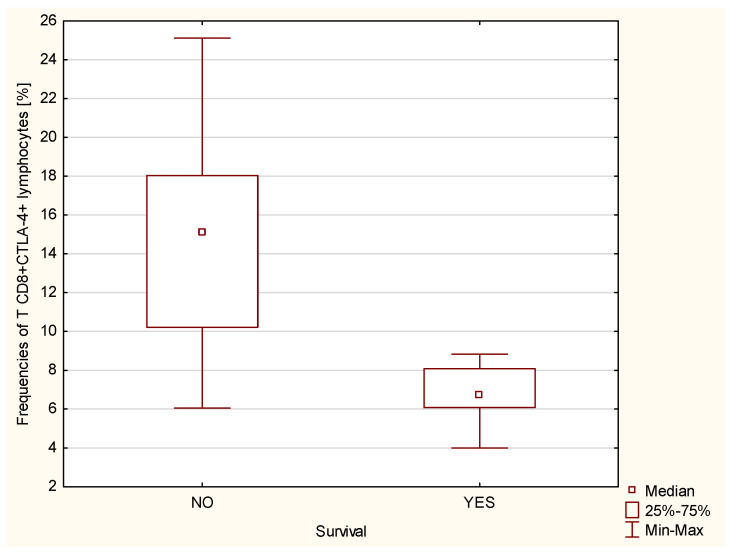
Percentage of CD8+CTLA4+ T lymphocytes in iPAH patients taking into account the survival of patients with iPAH (*p* ≤ 0.001).

We have shown that statistically significantly higher percentages of CD4+CTLA-4+ (*p* ≤ 0.01, Figure 5) and CD8+ CTLA-4+ (*p* ≤ 0.001, Figure 6) T cells, measured at the time of IPAH diagnosis, were found in patients who died within 5 years of the diagnosis, which allows us to consider both of the above lymphocyte subpopulations as a negative prognostic/predictive factor in iPAH.

**Figure 7:**
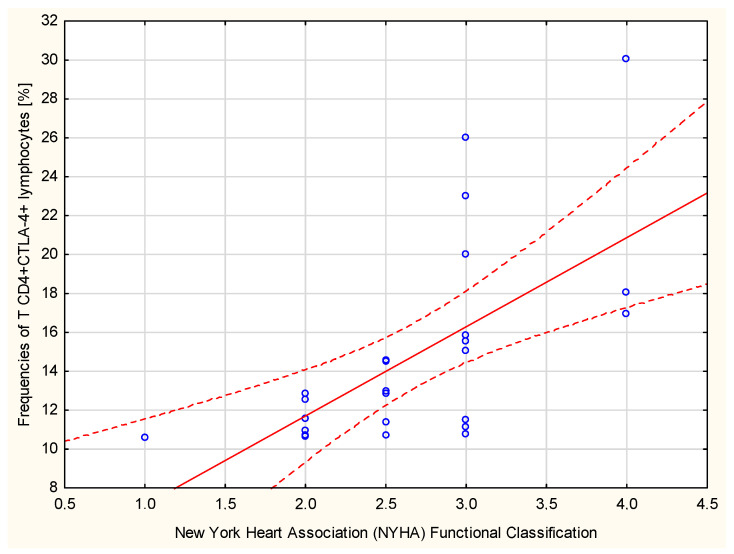
T CD4+CTLA4+ cells [%] and New York Heart Association (NYHA) Functional Classification; Spearman’s rank correlation r = 0.7077, *p* < 0.001.

The percentages of CD4+CTLA-4+ T cells correlated strongly positively with the severity of heart failure New York Heart Association (NYHA) Functional Classification (Spearman’s rank correlation r = 0.7077, *p* < 0.001, Figure 7).

**Figure 8:**
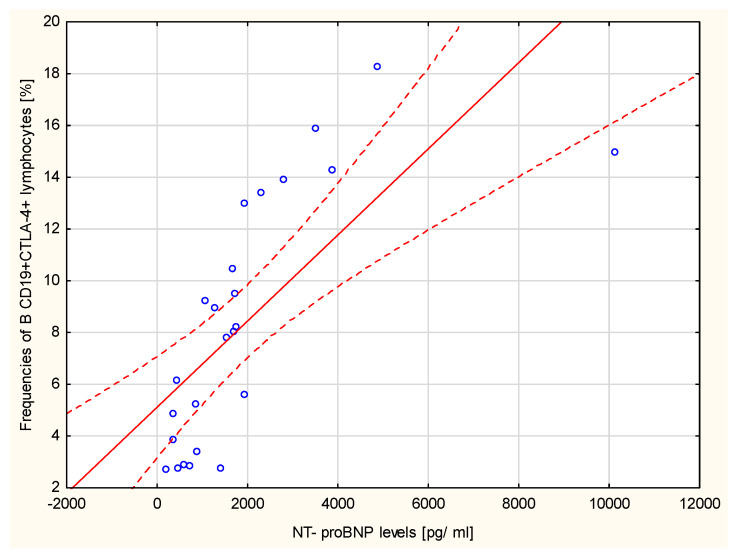
B CD19+CTLA–4+ cells [%] and NT–proBNP concentration [pg/mL]; Spearman’s rank correlation r = 0.8498, *p* < 0.001.

Moreover, the percentage of B CD19+CTLA-4+ cells strongly positively correlated with the concentration of NT-proBNP (r = 0.8498, *p* < 0.001, Figure 8).

## 3. Discussion

In this study, we analyzed lymphocyte subpopulations—CD4+, CD8+, CD19+—and surface antigen CTLA-4 in patients with different types of PAH: CHD-PAH, CTD-PAH, CTEPH and iPAH. Accordingly, CD4+, CD8+ and CD19+ levels were studied mostly in patients with iPAH. There are few data on patients with CHD-PAH, CTD-PAH and CTEPH so our study also focused on other types of PAH. CHD-PAH occurs in 5–10% of congenital heart disease (CHD) patients, mostly woman [11]. CTD-PAH is most common in patients with systemic scleroderma and its development contributes to poor disease prognosis and an increased risk of death [12]. CTEPH is a relatively rare type of PAH, possibly due to the great difficulty in diagnosis [13].

We focused on CTLA-4, which is a receptor on the surface of lymphocytes. This was because CTLA-4 controls T cell responses, alongside manipulation of CTLA-4, has become a cornerstone in the development of therapies for autoimmune diseases and cancer [14]. In general, CTLA-4 is a widely studied antigen for the treatment of malignancies; however, the close association of CTLA-4 blockade with the development of immune toxicity is problematic. The use of anti-CTLA4 blocking antibody has the effect of increasing Th17 cells in patients with metastatic melanoma, which enhances immune toxicity [15].

In our study, we observed a twofold increase in CD4+CTLA-4+ in patients with iPAH, but a decrease in patients with CTD-PAH and CTEPH. In CD4+ studies without CTLA-4, an increase in CD4+ T cells was reported in patients with PAH [16,17]. CD4+ T lymphocytes aggravate PAH progression, increase inflammation and exert autoimmune effects through the secretion of cytokines IL-2, IL-4, IL-6, IL-13, IL-21, TNF-α and IFN-γ by CD4+ T cells [18]. It was reported that CTLA-4 expression levels were elevated on activated Th cells in iPAH [19]. Moreover, an increased percentage of cTfh-17 cells in the CD4+ population was observed in patients with iPAH [20]. A study by Maston et al. [21] in mouse models showed that CD4+ cells have a role in the development of hypoxia induced by PAH. Additionally, in normoxic and CH mice, Th17 was present in the cells, along with increased levels of pro-inflammatory IL-6. This suggests that T cells have a role in PAH induction [21]. In CD4+ T cells cultured in the presence of monocyte-derived DCs (MoDCs) from patients with PAH, reduced expression of IL-4 (Th 2 response) and higher levels of IL-17 (Th17 response) and increased activation and proliferation of CD4+ T cells were observed, as compared with CD4+ T cells cultured with MoDCs from control patients [22]. In current literature, an increase in Treg levels in iPAH patients has been reported [23,24]. Sada et al. [25] concluded in a study of Treg cells in iPAH patients that CTLA-4 expression levels in the immunosuppressive CD_4_CD_45_RA+-FoxP3^high^ aTregs (aTregs) and CD_4_CD_45_RA+-FoxP3^low^ non-Tregs (non-Tregs) subgroups were higher than those in control patients; however, the level of aTregs subgroup in iPAH patients did not change when compared with healthy patients, and the level of non-Tregs subgroup was higher than in healthy patients [25]. In addition, Tm levels were increased in iPAH patients [24]. Our data show differences in CD4+CTLA-4+ levels in patients with iPAH versus CTD-PAH and CTEPH. This information sheds new light on previous studies. Because decreased CD4+CTLA-4+ levels in patients with CTD-PAH and CTEPH may correlate with the development of immune toxicity and, therefore, a severe disease course, we suggest further studies of the CD4+CTLA+ group as divided into CD4+ Th, Treg and Tm cells. Such work may provide the information needed to understand the mechanisms involved in CD4+ in patients with PAH.

In this study, we observed an increase in CD8+CTLA4+ T lymphocytes. We thus conclude that CD8+ T lymphocytes aggravate PAH progression, increase inflammation and exert autoimmune effects, albeit through strong cytolytic activity [26]. However, in a study by Hautefort et al. [22], no changes in CD8+ counts were found in patients with PAH. Still, an increase in CD8+ levels in patients with iPAH was reported [16,27] and Ulrich et al. [23] reported a decrease in CD8+ levels. In contrast, the percentage of CD8+ T cells was much higher than other T cells, and it seems that the inflammatory infiltrate in PAH consisted mainly of CD8+ [17,28]. A role for Tc in autoimmunity in PAH has been suggested based on information gleaned from tumor studies [23].

An absence of changes in CD19+ B lymphocytes levels in patients with PAH had been reported [20,22]. In our study, however, we observed an increase in CD19+CTLA-4+ in patients with iPAH, and a decrease in patients with CHD-PAH and CTD-PAH. The elevated levels of CTLA-4 found on B lymphocytes is an interesting observation because, under conditions of body equilibrium, CTLA-4 is not locatable on B lymphocytes [29]. Since CTLA-4 can appear on the surface of B lymphocytes as a result of activation by T lymphocytes [30], the elevated levels of CD19+CTLA-4+ may be a response to enhanced levels of CD4+CTLA-4+ and CD8+CTLA-4+ [31].

### Limitations of the Study

The limitation of the study is that it involves a small study group. The enrollment to the study was quite difficult because PAH is a rare disease and we only selected newly diagnosed PAH patients using quite strict inclusion criteria, such as no infection three months prior to the study, being without immunomodulatory treatment, no presence of allergy, etc. We only found 25 iPAH patients fulfilling the parameters. A larger study group may, therefore, provide more statistically significant correlations or differences between PAH patients and healthy controls.

## 4. Material and Methods

The study was conducted on 70 patients with PAH (50 women and 20 men). The diagnosis of PAH was based on ESC/ERS Guidelines [32]. The age of the patients was on average 57.74 ± 17.17 years (median: 60 years, minimum: 23 years, maximum: 81 years). Patients were classified by type of pulmonary arterial hypertension into chronic thromboembolic pulmonary hypertension (CTEPH) (10 patients, 7 women), PAH associated with congenital heart disease (CHD-PAH) (26 patients, 19 women), pulmonary arterial hypertension associated with systemic connective tissue disease (CTD-PAH) (9 patients, 9 women), and idiopathic pulmonary arterial hypertension (iPAH) (25 patients, 15 women). The heritable PAH patients were not included in this study. In patients with PAH, the WHO functional class of heart failure was established. The basic clinical and laboratory parameters characterizing patients with selected types of PAH and persons from the control group are decribed in Table 2. The basic hemodynamic parameters assessed during cardiac catheterization and echocardiography in patients with CHD-PAH, CTD-PAH, CTEPH and iPAHare delineated in Table 3.

The study was conducted in subjects who showed no signs of infection or allergy and did not have immunosuppressive treatment or a blood transfusion in the 3 months prior to the study.

The control group consisted of 20 subjects (12 women and 8 men) aged 58.1 ± 11.1 years (median: 56 years; minimum: 39 years; maximum: 77 years). Only subjects with no history of cardiovascular disease, no history of treatment with agents affecting the immune system, no history of infection, no history of autoimmune disease, no history of allergy and no history of blood transfusion were selected as volunteers.

The protocol of the conducted study received a positive opinion of the Bioethics Committee at the Medical University of Lublin (number KE-0254/309/2016). The material for the study was peripheral blood, which was collected from patients with pulmonary arterial hypertension and from the control group. Accordingly, 10 mL of blood was collected into tubes containing EDTA via an aspiration-vacuum system (Sarstedt, Germany). The collected blood was immediately processed to obtain plasma, to evaluate lymphocyte immunophenotypeand to isolate peripheral blood mononuclear cells (PBMCs).

### 4.1. Cytometric Analysis

Cytometric analysis was performed using CellQuest software (Becton Dickinson, Franklin Lakes, NJ, USA). The employment of a FACSCalibur flow cytometer (Becton Dickinson, USA) equipped with an argon laser (wavelength 488 nm) allowed for the reading of the following parameters: FSC, SSC, FL-1 (green fluorescence intensity), FL-2 (orange fluorescence intensity) and FL-3 (red fluorescence intensity). Herein, fluorescence intensity is dependent on antigen binding by monoclonal antibodies labeled with the appropriate fluorochromes.

Lymphocyte subpopulation and surface antigen analysis was performed with 20,000 cells counted from the lymphocyte gate (R1 region). The correct position of the gate was confirmed by using antibodies directed to CD45 and CD14 antigens. The result of the cytometric analysis was presented as the percentage of cells positively stained with the respective monoclonal antibodies.

To assess the presence of peripheral blood lymphocyte surface antigens, the appropriate monoclonal antibodies were separated into tubes at 20 µL. Subsequently, 50 µL of whole blood was added to each tube and the monoclonal antibodies were incubated with whole blood for 20 min at room temperature. Table 4 shows the monoclonal antibodies used for labeling and lists the fluorochromes to which they were conjugated.

### 4.2. Statistical Analysis

Descriptive characteristics of continuous variables were presented as: arithmetic mean, standard deviation (SD), minimum value, maximum value and median. Intergroup comparisons were performed using analysis of variance (ANOVA) with Duncan’s or Games–Howell post-hoc tests, depending on verification of the assumptions of analysis of variance, or the Kruskal–Wallis test with Dunn’s post-hoc test. Comparison of mean values of independent variables depended on meeting the criteria of normality of distributions and equality of variance was performed using Student’s *t* tests for independent samples.

## 5. Conclusions

Patients in the iPAH group had the significantly highest percentage of CD4+CTLA-4+ T lymphocytes among all PAH groups, as compared to those in the control group, patients with CTEPH, CTD-PAH and CHD-PAH.In iPAH patients, the percentages of CD4+CTLA-4+ T cells correlated strongly positively with the severity of heart failure New York Heart Association (NYHA) Functional Classification. Moreover, the percentage of B CD19+CTLA-4+ cells strongly positively correlated with the concentration of NT-proBNP. We have shown that statistically significantly higher percentages of CD4+CTLA-4+ and CD8+ CTLA-4+ T cells, measured at the time of iPAH diagnosis, were found in patients who died within 5 years of the diagnosis, which allows us to consider both of the above lymphocyte subpopulations as a negative prognostic/predictive factor in iPAH. CTLA-4 may be a promising biomarker of noninvasive detection of iPAH, but its role in planning the treatment strategy of PAH remains unclear. Further studies on T and B lymphocyte subsets are needed in different types of PAH to ascertain the relationships that exist between them and the disease. 

## Figures and Tables

**Table 1:** Evaluation of the percentage of CTLA-4 molecule in CHD-PAH, CTD-PAH, CTEPH and iPAH patients and controls.

Variable	Group	Median	Minimum	Maximum	Arithmetic Mean	SD	*p*
T-cells CD4+CTLA-4+ [%]	CHD-PAH	9.42	0.11	24.84	10.17	6.03	CHD-PAH vs. CTD-PAH (*p* ≤ 0.001), Control vs. CTD-PAH (*p* < 0.01), CHD-PAH vs. CTEPH (*p* < 0.01), CHD-PAH vs. iPAH (*p* < 0.01), Control vs. iPAH (*p* ≤ 0.001), CTD-PAH vs. iPAH (*p* ≤ 0.001), CTEPH vs. iPAH (*p* ≤ 0.001)
	CTD-PAH	2.04	0.53	2.4	1.8	0.6	
	CTEPH	3.91	2.57	5.4	3.98	1.02	
	iPAH	12.84	10.6	30.05	14.82	5.13	
	control	6.66	3.91	8.91	6.73	1.26	
T-cells CD8+CTLA-4+ [%]	CHD-PAH	7.49	1.4	16.32	8.18	3.85	CHD-PAH vs. control (*p* < 0.05), Control vs. iPAH (*p* ≤ 0.001)
	CTD-PAH	7.01	6.41	10.05	7.29	1.14	
	CTEPH	6.98	4.18	9.85	6.66	1.95	
	iPAH	8.33	4	25.11	10.58	6	
	control	5.46	1.36	8.54	5.19	1.87	
B-cells CD19+CTLA-4+ [%]	CHD-PAH	3.72	0.59	11.37	4.88	2.91	CHD-PAH vs. iPAH (*p* < 0.05), CTD-PAH vs. iPAH (*p* < 0.05),
	CTD-PAH	4.04	2.61	4.89	3.92	0.88	
	CTEPH	5.92	2.89	9.03	6.19	2.2	
	iPAH	8.01	2.69	18.28	8.34	4.8	
	control	5.68	2.48	8.47	5.35	1.92	

**Table 2:** Basic clinical and laboratory parameters characterizing patients with selected types of PAH and persons from the control group.

Parameter	Group	Median	Minimum	Maximum	Mean	SD	*p*
Age	CHD-PAH	57.5	23	81	55.69	17.34	CTEPH vs. CHD-PAH (*p* < 0.05), CTEPH vs. Control group (*p* < 0.05), CTEPH vs. CTD-PAH (*p* < 0.05), iPAH vs. CTEPH (*p* < 0.05),
	CTD-PAH	54	28	77	52.22	18.69	
	CTEPH	72.5	54	81	71.1	8.85	
	iPAH	62	23	81	56.52	17.23	
	Control group	56	39	77	58.05	11.12	
BMI	CHD-PAH	24.91	19.5	38.15	25.54	4.18	-
	CTD-PAH	22	20.32	27.98	23.79	2.96	
	CTEPH	23.67	20.44	35.04	24.74	4.18	
	iPAH	26	17.1	40.52	27.53	5.78	
	Control group	-	-	-	-	-	
6MWT [m]	CHD-PAH	378	50	578	323.15	149.64	-
	CTD-PAH	420	80	577.5	382.17	149.99	
	CTEPH	358.5	190	561	356.1	110.94	
	iPAH	374	136	556	377.84	99.38	
	Control group	-	-	-	-	-	
Neutrophilscount [10^3^/mm^3^]	CHD-PAH	4.55	2.3	9.69	4.64	1.82	-
	CTD-PAH	4.29	2.14	7.91	4.8	1.7	
	CTEPH	4.64	1.74	9.01	5.01	2.24	
	iPAH	5.11	2.08	8.43	5.16	1.64	
	Control group	3.94	2.71	6.03	4.32	1.03	
Lymphocytes count [10^3^/mm^3^]	CHD-PAH	1.68	1.1	2.77	1.72	0.46	Control group vs. CHD-PAH (*p* ≤ 0.001), CTD-PAH vs. CHD-PAH (*p* ≤ 0.001), iPAH vs. CHD-PAH (*p* < 0.05), iPAH vs. CTD-PAH (*p* < 0.05)
	CTD-PAH	2.6	1.67	3.04	2.47	0.4	
	CTEPH	2.42	1.3	3.83	2.51	0.96	
	iPAH	2.01	1.2	3.14	2.15	0.56	
	Control group	2.54	1.53	3.07	2.44	0.45	
Hemoglobin concentration [g/dL]	CHD-PAH	15.1	7.4	22.1	15.4	4.38	-
	CTD-PAH	13.5	11.3	19.4	13.76	2.42	
	CTEPH	13.75	8.5	16.7	13.24	2.65	
	iPAH	13.6	9.5	18.5	13.69	2.04	
	Control group	14.35	12.5	15.6	14.31	0.86	
Platelets count [mm^3^]	CHD-PAH	156,500	62,000	299,000	164,115	64,380	Control group vs. CHD-PAH (*p* ≤ 0.001), CTD-PAH vs. CHD-PAH (*p* < 0.05), iPAH vs. CHD-PAH (*p* < 0.05), CTD-PAH vs. Control group (*p* ≤ 0.001), CTEPH vs. Control group (*p* < 0.01), iPAH vs. Control group (*p* < 0.01), CTEPH vs. CTD-PAH (*p* < 0.05), iPAH vs. CTD-PAH (*p* ≤ 0.001)
	CTD-PAH	114,000	55,000	309,000	147,889	92,369	
	CTEPH	182,500	93,000	348,000	189,000	73,138	
	iPAH	213,000	78,000	474,000	213,800	80,647	
	Control group	262,500	186,000	344,000	263,950	52,744	
AspAT [U/L]	CHD-PAH	20	12	68	25.46	13.95	-
	CTD-PAH	32	17	38	27.67	7.79	
	CTEPH	27	19	127	36.4	32.22	
	iPAH	22	10	49	23.12	9.18	
	Control group	22.5	13	34	22.6	6.1	
ALAT [U/L]	CHD-PAH	16	26	22	22	16.87	Control group vs. CHD-PAH (*p* < 0.05), CTEPH vs. CHD-PAH (*p* < 0.01), CTEPH vs. CTD-PAH (*p* < 0.05), iPAH vs. CTEPH (*p* < 0.05)
	CTD-PAH	18	9	25.78	25.78	21.72	
	CTEPH	22.5	10	29.8	29.8	22.05	
	iPAH	18	25	20.04	20.04	10.41	
	Control group	18.5	20	19.75	19.75	7.49	
T CD3+ lymphocytes [%]	CHD-PAH	71.3	60.29	80.9	70.68	6.31	-
	CTD-PAH	70.53	57.31	80.45	70.29	6.85	
	CTEPH	69.8	63.1	81.62	71.07	6.3	
	iPAH	70.82	59.21	89.16	71.11	6.84	
	Control group	68.08	60.63	74.49	68.26	3.84	
B CD19+ lymphocytes [%]	CHD-PAH	9.36	2.46	26.25	9.76	4.89	
	CTD-PAH	9.05	2.79	21.99	10.18	6.29	
	CTEPH	11.25	5.36	16.91	11.01	4.14	
	iPAH	11.9	3.5	20.67	11.36	4.97	
	Control group	11.39	6.04	16.9	11.25	2.5	
NK cells (CD3-/CD16+CD56+) [%]	CHD-PAH	22.37	6.63	37.5	20.88	7.97	Control group vs. CHD-PAH (*p* < 0.05). CTD-PAH vs. CHD-PAH (*p* ≤ 0.001). CTEPH vs. CHD-PAH (*p* ≤ 0.001). iPAH vs. CHD-PAH (*p* ≤ 0.001). CTD-PAH vs. Control group (*p* ≤ 0.001). CTEPH vs. Control group (*p* < 0.05). iPAH vs. Control group (*p* ≤ 0.001)
	CTD-PAH	10.9	2.34	19.77	10.82	5.76	
	CTEPH	8.93	4.32	17.41	9.65	4.6	
	iPAH	11.23	3.99	20.43	11.1	4.22	
	Control group	14.43	12.16	19.34	15.35	2.25	
NKT-like cells CD3+CD16+CD56+ [%]	CHD-PAH	1.58	0.24	8.47	2.62	2.31	CHD-PAH vs. iPAH (*p* < 0.01)
	CTD-PAH	1.04	0.21	8.2	2.97	3.08	
	CTEPH	3.17	0.77	11.26	4.1	3.68	
	iPAH	5.23	0.67	10.94	5.26	2.67	
	Control group	3.27	1.15	4.92	3.02	1.02	
T CD4+/CD3+ lymphocytes [%]	CHD-PAH	42.16	21.58	57.43	41.25	10	CTD-PAH vs. Control group (*p* < 0.01). CTEPH vs. CTD-PAH (*p* < 0.01)
	CTD-PAH	35.54	28.43	59.88	39.84	10.22	
	CTEPH	45.37	39.14	51.33	45.42	4.47	
	iPAH	36.91	19.73	62.92	38.68	13.42	
	Control group	44.16	40.71	48.84	44.46	2.5	
T CD8+/CD3+ lymphocytes [%]	CHD-PAH	27.21	12.78	47.16	26.94	8.2	Control group vs. CHD-PAH (*p* ≤ 0.001). CTEPH vs. Control group (*p* < 0.01). iPAH vs. Control group (*p* ≤ 0.001)
	CTD-PAH	30.62	10.18	39.87	28	10.82	
	CTEPH	20.29	11.17	36.94	23.02	7.67	
	iPAH	28.3	9.19	59.29	29.46	14.17	
	Control group	34.73	29.33	39.6	34.36	3.29	
T CD4+: T CD8+ lymphocytes ratio	CHD-PAH	1.62	0.46	4.49	1.78	0.95	Control group vs. CTEPH (*p* < 0.05), CTD-PAH vs. CTEPH (*p* < 0.05)
	CTD-PAH	1.31	0.87	4.86	1.84	1.38	
	CTEPH	2.09	1.09	4.5	2.24	0.97	
	iPAH	1.25	0.34	6.85	1.95	1.78	
	Control group	1.29	1.03	1.57	1.31	0.16	
T regulatory cells [%]	CHD-PAH	7.43	4.67	15.59	8.43	2.87	CHD-PAH vs. CTEPH (*p* < 0.01), Control group vs. CTEPH (*p* < 0.05), CTD-PAH vs. CTEPH (*p* < 0.05), CHD-PAH vs. iPAH (*p* < 0.01), Control group vs. iPAH (*p* ≤ 0.001), CTD-PAH vs. iPAH (*p* < 0.05), TEPH vs. iPAH (*p* ≤ 0.001)
	CTD-PAH	7.25	4.73	11.59	8.11	2.39	
	CTEPH	4.13	1.79	10.33	4.65	2.55	
	iPAH	11.21	5.94	23.81	11.98	3.97	
	Control group	7.37	3.15	10.15	7.1	1.94	
NT-proBNP [pg/mL]	CHD-PAH	836	106.8	9350	1597.66	1930.48	-
	CTD-PAH	1279	429	4015	1530	1384.07	
	CTEPH	1756.5	53	5991	2071.52	1617.33	
	iPAH	1546	210	10,144	1940.22	2072.29	
	Control group	-	-	-	-	-	

6MWT: 6-min walk test; ALAT: alanine transaminase; AspAT: aspartate transaminase; BMI: body mass index; CHD-PAH: pulmonary arterial hypertension associated with congenital heart disease; CTD-PAH: pulmonary arterial hypertension associated with connective tissue disease; CTEPH: chronic thromboembolic pulmonary hypertension; iPAH: idiopathic pulmonary arterial hypertension; NK: natural killer; SD: standard deviation.

**Table 3:** Basic hemodynamic parameters assessed during cardiac catheterization and echocardiography in patients with CHD-PAH, CTD-PAH, CTEPH and iPAH.

Parameter	Group	Median	Minimum	Maximum	Mean	SD	*p*
Pulmonary vascular resistance (PVR) [dyne/s/cm^−5^	CHD-PAH	838.62	134	2803	1066.32	696.02	CHD-PAH vs. CTD-PAH (*p* ≤ 0.001). CTD-PAH vs. CTEPH (*p* < 0.05). CTD-PAH vs. iPAH (*p* < 0.05)
	CTD-PAH	355	139	1292	424.53	369.55	
	CTEPH	715.5	305.51	1125.8	720.4	234.12	
	iPAH	651	158	1599	697.6	314.62	
	Control group	-	-	-	-	-	
Cardiac index (CI) [L/min/m^2^]	CHD-PAH	2.27	1.65	7.32	2.52	1.08	CTEPH vs. CHD-PAH (*p* < 0.05). CTEPH vs. CTD-PAH (*p* < 0.05). iPAH vs. CTEPH (*p* < 0.01)
	CTD-PAH	3.21	1.83	4.67	3.16	0.83	
	CTEPH	2.05	1.75	5.8	2.53	1.25	
	iPAH	2.6	1.43	3.75	2.54	0.65	
	Control group	-	-	-	-	-	
Cardiac output (CO) [L/min]	CHD-PAH	3.84	2.29	13.9	4.2	2.11	CTD-PAH vs. CHD-PAH (*p* ≤ 0.001). iPAH vs. CHD-PAH (*p* < 0.01). CTEPH vs. CTD-PAH (*p* < 0.01). iPAH vs. CTEPH (*p* < 0.05)
	CTD-PAH	5.75	3.02	8.47	5.37	1.57	
	CTEPH	3.56	2.48	9.51	4.19	2.17	
	iPAH	4.46	2.11	6.42	4.64	1.15	
	Control group	-	-	-	-	-	
Mean right atrial pressure (mRAP) [mmHg]	CHD-PAH	8	1	16	7.88	3.25	-
	CTD-PAH	8	3	15	8.56	4.03	
	CTEPH	9	3	18	9.5	5.02	
	iPAH	9	2	23	8.8	5.69	
	Control group	-	-	-	-	-	
Mean pulmonary artery pressure (mPAP) [mmHg]	CHD-PAH	48.5	26	106	53.61	22.76	CHD-PAH vs. CTD-PAH (*p* < 0.01). CTD-PAH vs. CTEPH (*p* < 0.05). CTD-PAH vs. iPAH (*p* < 0.05)
	CTD-PAH	34	25	68	35.3	13.25	
	CTEPH	46	25.6	56	45.66	8.75	
	iPAH	48	25	66	45.56	12.03	
	Control group	-	-	-	-	-	
Mean pulmonary artery pressure by echocardiography (PASP) [mmHg]	CHD-PAH	76.5	41	150	82.15	29.02	CTD-PAH vs. CHD-PAH (*p* < 0.01). CTEPH vs. CTD-PAH (*p* < 0.05). iPAH vs. CTD-PAH (*p* < 0.05)
	CTD-PAH	57	35	110	58.22	21.68	
	CTEPH	78.5	39	110	80.4	21.57	
	iPAH	77	37	105	72.2	18.92	
	Control group	-	-	-	-	-	
Mean pressure in the right ventricle on echocardiography [mmHg]	CHD-PAH	72	40	150	81.19	29.69	CHD-PAH vs. CTD-PAH (*p* < 0.05). CTD-PAH vs. CTEPH (*p* < 0.05).
	CTD-PAH	56	36	115	58.67	24.07	
	CTEPH	76.5	50	110	79.7	18.54	
	iPAH	76	42	96	68.84	17.66	
	Control group	-	-	-	-	-	

CHD-PAH: pulmonary arterial hypertension associated with congenital heart disease; CTD-PAH: pulmonary arterial hypertension associated with connective tissue disease; CTEPH: chronic thromboembolic pulmonary hypertension; iPAH: idiopathic pulmonary arterial hypertension; SD: standard deviation.

**Table 4:** List of antibodies used to assess lymphocyte immunophenotype.

Structure	Antibody	Fluorochrome
T-cells	anty–CD4	FITC
T-cells	anty–CD8	FITC
B-cells	anty–CD19	FITC
CTLA-4	anty–CTLA-4	PE-Cy5

## Data Availability

Due to privacy and ethical concerns, the data that support the findings of this study are available on request from the First Author, (M.T.).
